# Does national forest city improve residents' health? Evidence from China

**DOI:** 10.3389/fpubh.2024.1304600

**Published:** 2024-02-20

**Authors:** Hanjin Xie, Chunmei Yang, Xi Tan

**Affiliations:** ^1^School of Economics and Management, East China Jiaotong University, Nanchang, China; ^2^School of Economics and Management, Wuhan University, Wuhan, China

**Keywords:** national forest city, residents' health, health risk exposure, health behaviors, mental health

## Abstract

**Objective:**

National health is essential for economic and social development. The aim of this article is to examine the relationship, heterogeneity effects and influential mechanisms between National Forest Cities and the residents' health.

**Methods:**

The article matches the China Family Panel Studies data in 2018 (CFPS2018) with the 2016–2018 National Forest Cities Construction List, resulting in a final sample of 20,041. Oprobit, Ologit, Instrumental Variable technique (2SLS) and interaction term analysis were used as the main research methods in this article.

**Results:**

The findings indicate that: (1) The construction of National Forest Cities significantly improves the residents' health in terms of both physical and mental health, and this conclusion is still valid after a series of robustness tests. (2) On the one hand, National Forest Cities promote residents' health by reducing air pollutants such as SO_2_ and soot to reduce residents' health risk exposure; On the other hand, it promotes residents' health by positively guiding them to engage in healthy behaviors. (3) National Forest Cities have a greater effect on the health of urban residents, older adult and lower-income group, suggesting that National Forest Cities are a public benefit.

**Conclusions:**

The construction of National Forest Cities is a public welfare that promotes residents' health, and it is an important revelation for accelerating the realization of the Healthy China Strategy. The article provides new empirical evidence for understanding the welfare effects of forest cities and offers new practical paths for improving residents' health.

## 1 Introduction

In recent years, the pace of people's lives has accelerated due to the accelerated process of urbanization, population aging and changing lifestyles. A life of daily labor and emotional stress has left more and more people in a state of sub-health, which has led to the outbreak of a series of “civilization diseases” and “chronic diseases.” According to the 2020 Report on Nutrition and Chronic Disease Status of Chinese Residents, deaths due to chronic diseases accounted for 88.5% of total deaths in China in 2019, the prevalence of depression reached 2.1%, and the prevalence of anxiety disorders was 4.98%. Analyzing such reports, it is clear that there is a general demand for “preventing illness before it occurs.” People are gradually paying more attention to this, and more and more people aspire to go to the ecologically sound environment for recreation and health care. Urban forests, as the most unique resource, provide a wide range of ecosystem services to humans and can help combat many urban ills (1).

Reviewing previous studies, factors affecting human health are numerous and complex, including income status (2–7), education level (8), healthcare services (9), lifestyle (10), and health behaviors (11). Scholars have also found heterogeneity in the effects of some factors on health, such as the significantly higher mortality effect of alcohol consumption on lower class groups than on higher class groups (12), and the fact that if women's physical activity during labor and leisure time is comparable to men's, then women over the age of 54 will be healthier than men (13). Furthermore, in addition to economic factors and individual behavior that can have an impact on health, the environment can have a significant impact on health. Some studies have shown that environmental pollution can jeopardize health by increasing the incidence of various diseases (14), such as worsening the condition of respiratory diseases (15). Moreover, environmental pollution also harms mental health by increasing residents' anxiety and despair, further inhibiting residents' life satisfaction and subjective wellbeing (16–18), and even increasing the risk of suicide (19). Green spaces can reduce regional population mortality to some extent (20, 21) and have positive effects on negative states such as depression (22) and anxiety (23). However, at the same time, there are a few studies with different conclusions about the relationship between green spaces and health. For example, it was found that there is a lack of clear association between urban green space and health in Oslo (24). A similar study in New Zealand found no association between green space and mortality from cardiovascular disease or lung cancer (25). Galina Chukina, a senior researcher at the Institute for Advanced Sustainability Studies in Potsdam, Germany, who studies emissions from urban trees, said: when a street is lined with large plantings of certain tree species, those trees can significantly increase ozone levels there, and high near-ground-level concentrations of ozone can trigger asthma, bronchitis and other respiratory illnesses.

Therefore, there is uncertainty as to whether the construction of National Forest Cities (NFCs) will improve the residents' health. On the one hand, restorative environments can increase one's positive emotions (26, 27), release one's anxiety and stress (28, 29), improve one's self-identity (30), and stimulate one's creativity (31), all of which are important determinants of good health. On the other hand, In order to obtain more green space within the constraints of limited land resources, the State will exercise more control over land supply, and the end result may be less land for other uses, such as some commercial land. In both cases, urban green space strategies may be self-contradictory. While the creation of new green space can ameliorate environmental problems (32, 33), it may also increase housing costs and property values, which brings precarious housing conditions with negative public health implications (34, 35). Ultimately, this may lead to the fact that the impact of NFCs on the residents' health is uncertain, with the net effect depending on the relative strength of the two effects. Therefore, we need to clarify the ultimate effect of NFCs on the residents' health.

## 2 Materials and methods

### 2.1 Introduction to the national forest city indicator system

The NFCs Initiative was launched by the Chinese Government, which refers to cities whose urban ecosystems are dominated by forest vegetation, where urban ecological construction realizes the integrated development of urban and rural areas, and where the construction indicators meet the requirements of the relevant standards, and which are ultimately approved by State Forestry Administration. The NFCs Evaluation Indicators contain seven specific aspects, including comprehensive indicators, coverage rate, forest ecological network, forest health, public recreation, ecological culture, and rural greening, each of which has its own specific sub-criteria.

In recent years, the relevant regulations and management systems for the construction of NFCs have been continuously improved, especially in 2015, when forest cities were included in China's “13th 5-Year Plan.” In September 2016, the State Forestry Administration (PRC) issued the Guiding Opinions on Efforts to Develop Forest Cities and the Measures for the Approval and Management of the National Forest City Designation (draft for comments). All along, the NFC has always taken “let the forest come into the city, let the city embrace the forest” as its purpose. Doing well in “planting green in the earth” and “sowing green in the heart” has set a model and played a leading role in China's urban ecological construction.

### 2.2 Data

The data in this article comes from the “2018 China Family Panel Studies (CFPS2018)” conducted by the China Center for Social Science Surveys at Peking University, the list of NFCs published by the State Forestry Administration from 2016 to 2018, and the 2018 China Urban Statistical Yearbook. The CFPS data is a high-quality micro-database that focuses on the economic and non-economic wellbeing of China's population, including economic activity, educational attainment, family relationships and household dynamics, population migration, health, and more. This data was selected for this article because the CFPS data contains a module on the residents' health, which investigates the health status of the interviewed residents, thus providing data support for the study of the impact of the NFCs on the residents' health. We merged the data of individuals and households based on pid (personal ID), then matched the data of NFCs based on the county/district codes of the interviewed residents, and eliminated the observations of missing variable data, finally obtaining a sample of 20,041 analyses.

### 2.3 Variables

#### 2.3.1 Dependent variable

The dependent variable in this article is residents' health, following the prevailing practice in existing studies, using self-reported physical health (36, 37) and Center for Epidemiologic Studies Depression Scale (CESD) scores (38), which were used in this article as proxies for physical health and mental health, respectively.

The relevant question for the self-reported physical health in the CFPS2018 questionnaire setting is (qp201) “How healthy do you think you are?,” respondents were asked to choose a number between 1 and 5 according to their health status. Where “1” means pretty healthy, “5” means unhealthy, and so on in the middle. For ease of presentation and interpretation, the dependent variable (health) in this paper takes the value of 6 minus the value of the survey, so that in this paper 1 = unhealthy, 2 = average, 3 = relatively healthy, 4 = very healthy, and 5 = pretty healthy. Compared with a single measure, self-reported health is a comprehensive judgment of the respondent based on many factors such as disease severity, family history of disease, and stability of health status, and it is highly correlated with objective health such as personal morbidity and mortality (39). This measure has been widely used in studies because it meets psychometric adequacy, statistical reliability and validity (36, 37). In this article, self-reported health will be used as the main dependent variable. At the same time, other variables that can measure health will be used in this article for robustness tests.

CESD scores in the CFPS2018 questionnaire were set up with the question “Here are some feelings or behaviors that you may have experienced, please indicate how often each has occurred in the past week, based on your actual situation,” using the Center for Epidemiologic Studies Depression Scale containing eight questions as shown in Table 1. The eight questions shared the following options: hardly ever (less than a day), some of the time (1–2 days), often (3–4 days), most of the time (5–7 days), and were assigned values of 1–4, respectively. Since qn406, qn407, qn411, qn414, qn418, and qn420 are negative mood questions, i.e., the greater the value answered for these indicators, the more severe the depression. Therefore, the responses to the above questions were first positively normalized: 5 minus the value of the survey, and the converted value represented an increasing degree of mental health from 1 to 4. Finally, the scores of the above eight indicators were summed up to obtain the another dependent variable (mental), which ranges from 8 to 32, with larger scores representing better mental health.

**Table 1:** CESD scale.

**Question No**.	**Content of the question**	**Question No**.	**Content of the question**
qn406	I'm feeling down.	qn414	I feel alone.
qn407	I find it hard to get anything done.	qn416	I'm happy with my life.
qn411	I don't sleep well.	qn418	I feel sad.
qn412	I feel good.	qn420	I don't think life can go on.

#### 2.3.2 Independent variable

The independent variable (forestcity) is a dummy variable. It is equal to 1 if the location where the resident lives was the National Forest City in 2016–2018, and 0 otherwise.

#### 2.3.3 Instrumental variable

The endogeneity problem of the baseline model regression may come from two aspects: one is the omitted variable problem, and the other is the reverse causality problem. These problems are almost unavoidable in real life. Therefore, in this paper, the two-stage least squares (2SLS) method is used to test and analyze endogeneity, and the instrumental variable (IV) used is the number of National Forest Cities (NCities) in the province outside the city. This is because the greater the number of cities in a province that carry out the construction of NFCs, the higher the importance of the province to the construction of NFCs, and the greater the possibility of the city to carry out the construction of NFC, so the instrumental variable selected in this article meets the requirements of relevance. In addition, the construction of NFCs in other cities will not directly affect the residents' health in the city, so the instrumental variable meets the requirement of exogeneity.

#### 2.3.4 Control variables

In order to apply controls on the impact of other factors on residents' health, we introduced 13 variables at the individual, family, and city levels.

Nine control variables were introduced at the individual level, all from the CFPS 2018 adult questionnaire database, including an individual's gender (gender), age (age), marital status (marriage), urban/rural categorization (urban), whether or not they have ever smoked (smoking), highest level of educational attainment (edu), number of hours worked per week (work), Gross income from work (income), and frequency of exercise (exercise).

In terms of family level, we introduced two characteristics: household size (fsize), whether or not one owns home (house), all of which are taken from the CFPS 2018 household questionnaire database.

Finally, we also introduced two city characteristics: population size (pop), and public service level (hospital), with data from the China Urban Statistical Yearbook published by the National Bureau of Statistics. Table 2 describes the main variables.

**Table 2:** Description of main variables.

**Variable type**	**Variable name**	**Meaning**	**Description**
Dependent variables	Health	“How healthy do you think you are?”	1 = unhealthy, 2 = average, 3 = relatively healthy, 4 = very healthy, and 5 = pretty healthy
	Mental	CESD scores	Scores range from 8–32, with higher scores indicating better mental health
Independent variable	Forestcity	Whether it is a National Forest City for 2016–2018	1 = yes; 0 = no
Individual level control variables	Gender	Gender	1 = male; 0 = female
	Age	Age	Unit: years
	Marriage	Marital status	1 = unmarried; 2 = in marriage (with spouse); 3 = cohabiting; 4 = divorced; 5 = widowed
	Urban	Urban/rural classification	1 = urban; 0 = rural
	Smoking	Ever smoked	1 = yes; 0 = no
	Edu	Highest education received	0 = illiterate/semi-illiterate; 3 = elementary school; 4 = junior high school; 5 = high school/secondary school/technical school/vocational high school, 6 = junior college; 7 = undergraduate 7; 8 = master's degree; 9 = doctoral degree
	Work	Weekly working hours	Unit: hour
	Income	Income	Unit: Ten thousand yuan
	Exercise	Frequency of exercise	Unit: Times
Family level control variables	Fsize	Family Size	Unit: pcs
	House	Whether or not you own your own home	1 = yes; 0 = no
City level control variables	Pop	Population size (population density)	Unit: meter/person
	Hospital	Level of public services (logarithmic number of hospital beds)	Unit: pcs

#### 2.3.5 Mechanism variables

The construction of NFCs has increased the forest coverage and increased the construction of urban forest ecological network. On the one hand, forest belts can block air flow, which leads to the deposition of air particles (40, 41); on the other hand, plants can exchange gases with the outside world through stomata and lenticels, degrading harmful components in gases or storing them in organs (42, 43). Therefore, this paper verifies the impact mechanism from the perspective of reducing health risk exposure. Health risk exposure is measured by air quality indicators (PM_2.5_, CO_2_, SO_2_, and soot concentrations), and the relevant data are from the China Urban Statistical Yearbook.

In NFCs, there are a number of recreational green spaces, mainly various types of parks and public green spaces. On average, there is a recreational green space within an average of 500 meters away from most people's homes. In the outskirts of cities, there are forest parks and other types of ecotourism and recreation sites. Such recreational green spaces shorten the distance between residents and green spaces, and provide venues for residents' exercise activities (44). Therefore, it will motivate residents to engage in exercise behavior and improve physical function by promoting exercise (45). For example, Toftager et al. found that the frequency of physical activity was associated with distance to green space, and that people living more than 1 km from a green space were more likely to be obese (BMI ≥ 30) than those living < 300 m from a green space (46). Tamosiunas et al. found that the prevalence of cardiovascular disease (CVD) and its risk factors were related to the distance and use of urban green spaces, and that the prevalence of cardiovascular risk factors and diabetes was significantly lower in park users than in non-users (47). Therefore, this article verifies the impact mechanism from the perspective of promoting residents' health behavior. The frequency of residents' exercise in a week and the cumulative length of exercise in a week were used to characterize residents' health behaviors, and the relevant questions set in the CFPS2018 questionnaire were (qp701) “How many times did you exercise in the past week?” and (qp702) “In the past week, how long did you exercise in total?”

#### 2.3.6 Descriptive statistical analysis

Table 3 shows the means comparison of the health status variables for the NFCs and non-NFCs. The mean value of physical health is 2.906, indicating that the overall physical health of the residents is close to “relatively healthy.” The mean value of mental health is 26.359, indicating that most of the residents have a relatively optimistic attitude toward life. In terms of subgroups, the mean values of physical health and mental health in NFCs are slightly larger than those in non-NFCs.

**Table 3:** Comparison of means.

**Mean values**							
	**National Forest City**		**Non-National Forest City**		**All observations**		
**Variable**	**Mean**	**Sd**	**Mean**	**Sd**	**Mean**	**Sd**	**Obs**
Health	3.037	1.240	2.885	1.176	2.906	1.186	20,041
Mental	26.847	3.849	26.282	4.092	26.359	4.065	20,041
**Difference-in-means test**							
	**Levene's** ***T*****-test**		**Variance ratio test**		***T-*** **test**		
**Variable**	***T*** **-value**	***P-*** **value**	***F*** **-value**	***P*** **-value**	**Diff**	***T-*** **value**	***P-*** **value**
Health	4.23	0.0397	0.8984	0.000	−0.157	−6.213	0.000
Mental	15.32	0.0001	1.1213	0.000	−0.563	−7.061	0.000

In order to test whether the difference between the means of the above variables in NFCs and non-NFCs is statistically significant or not, this article does the *T*-test. Firstly, test for homoscedasticity, and then we choose whether to do the *T*-test for homoscedasticity or the *T*-test for heteroscedasticity. The results are shown in Table 3, the *P-*value of Levene's *T*-test and the *P*-value of Variance ratio test are < 0.05, which rejects the original hypothesis of homoscedasticity, indicating that the choice should be made to do the *T*-test of heteroscedasticity. The result of the heteroscedasticity *T*-test indicates that the difference in the means of this variable between the two samples is significant at 1% level.

**Figure 1:**
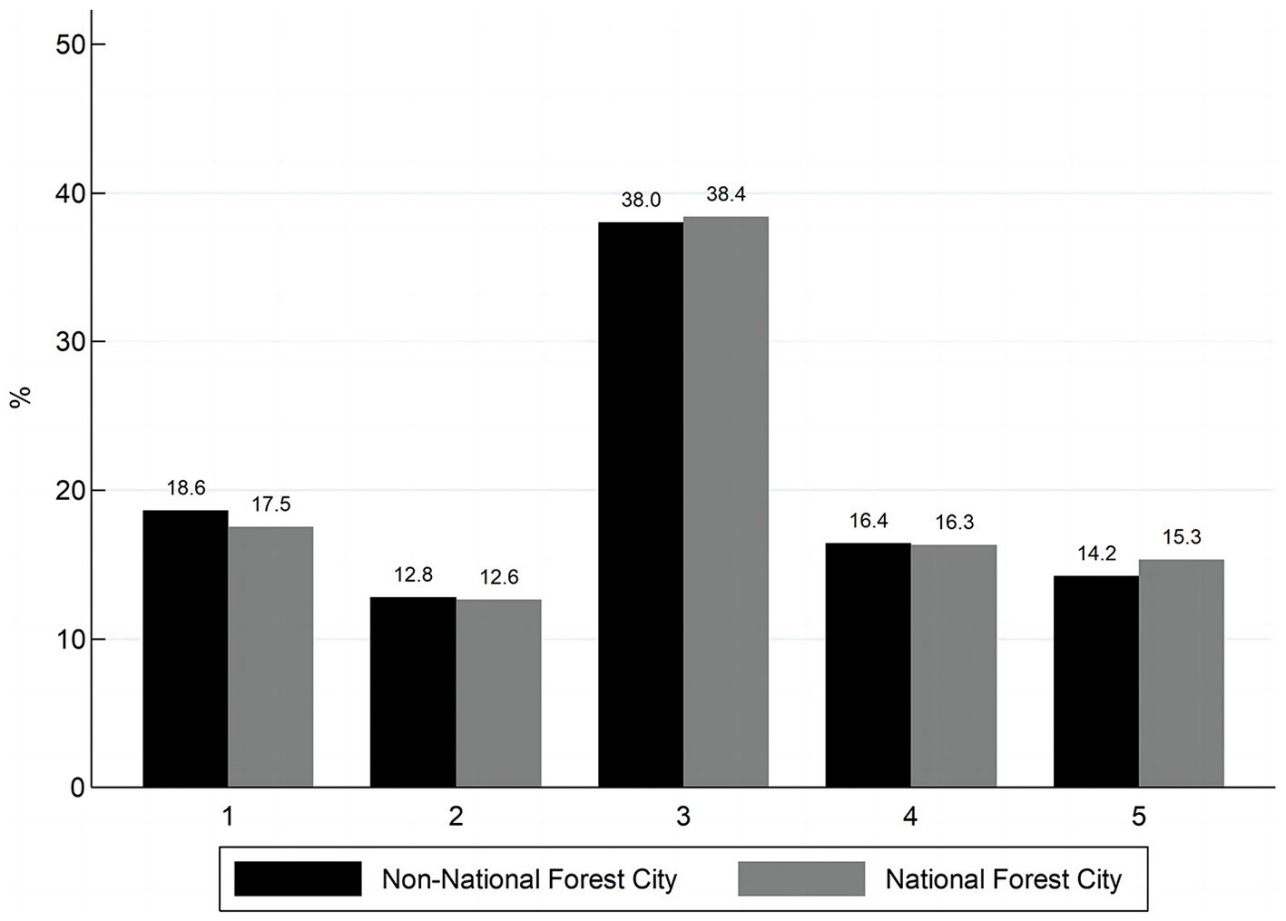
Distribution of physical health.

**Figure 2:**
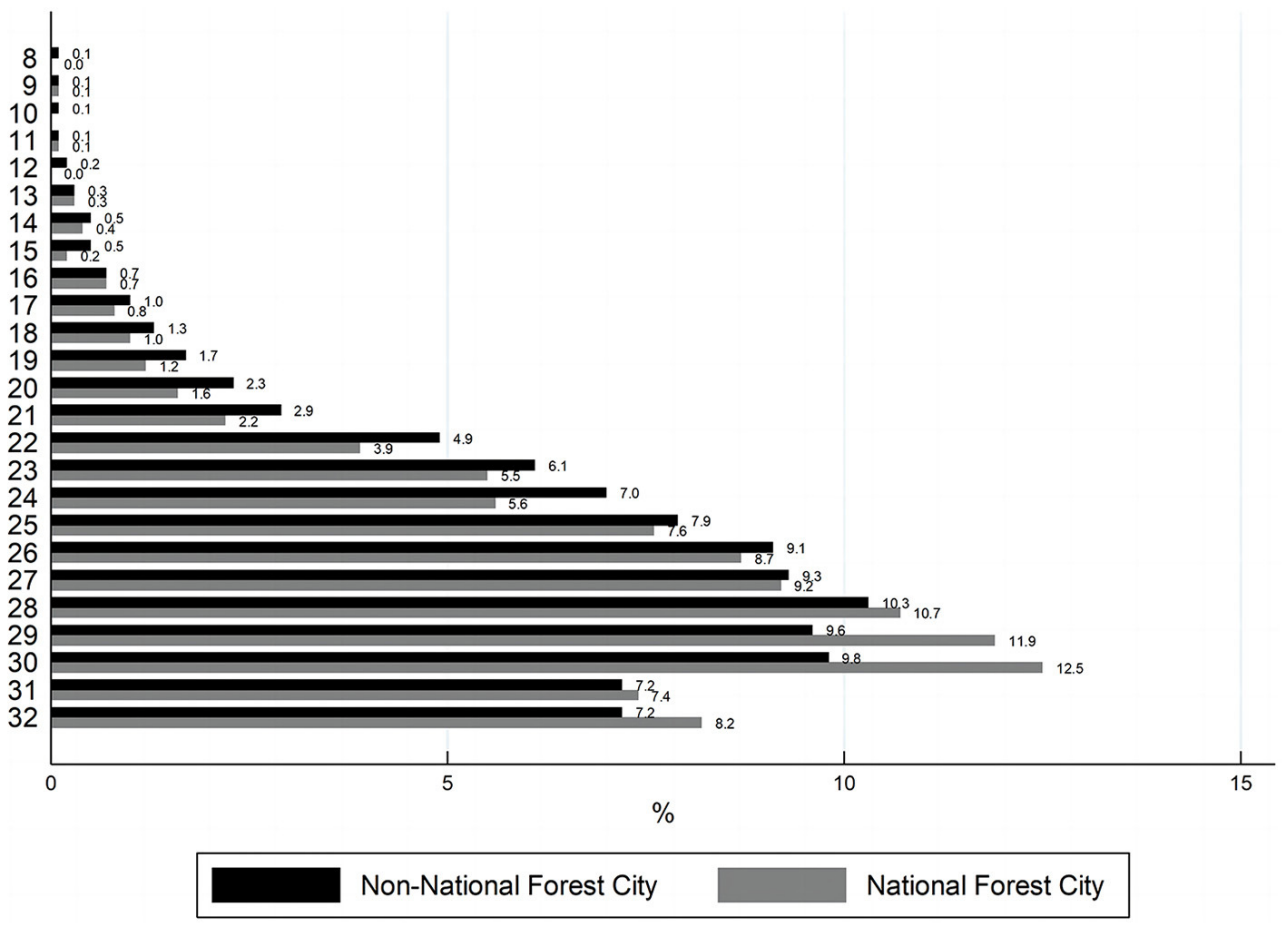
Distribution of mental health.

In order to visualize the differences between the two groups, the samples are divided into two groups according to whether they live in NFC or not. Figures 1, 2 compare the distributions of the physical health and mental health of the residents in these two groups respectively. It can be seen that residents of NFCs have a higher percentage of distribution on the “pretty healthy” and “healthy” options of residents' physical health than residents of non-NFCs. The proportion of residents in “average” and “unhealthy” is lower than that of residents in non-NFCs. The distribution on “relatively healthy” is similar for both groups. The distribution of residents' mental health also reflects a similar pattern, with NFCs accounting for a larger proportion of the regions with high scores. Therefore, we can tentatively conclude that NFCs promote the residents' health, but further empirical testing of the relationship between the two is still needed.

## 3 Results

### 3.1 Baseline model regression

The two dependent variables (health and mental) in this article are ordered data, and OLS estimation is not applicable here. So we use Ordered Probit and Ordered Logit models for regression estimation, which are specifically designed to deal with the situation where the dependent variable is ordered data. These two models are extensions of the Probit model and Logit model, which correspond to variables that are normally distributed and logistically distributed, respectively. Since there is not much difference between the two distributions when the sample size is relatively large, this paper applies both models to analyze the impact of NFCs on residents' health to ensure the robustness of the results.

The results are shown in Table 4. As can be seen from the regression results in column (1), after controlling the regional economic development level and other control variables, the results of the Oprobit model and Ologit model show that the coefficients of the NFCs are significantly positive at the 1% level, indicating that the construction of the NFCs contributes to the improvement of the residents' health on the whole. Meanwhile, it can also be seen from the regression results that the health of those who used to smoke is lower; and there is a significant positive correlation between the frequency of physical exercise and the residents' health, which is consistent with the actual situation.

**Table 4:** Baseline model regression.

	**(1) Oprobit**	**(2)** **Ologit**	**(3) Oprobit**	**(4)** **Ologit**
	**Health**	**Health**	**Mental**	**Mental**
Forestcity	0.137^***^ (0.024)	0.212^***^ (0.041)	0.143^***^ (0.021)	0.257^***^ (0.036)
Gender	0.155^***^ (0.020)	0.267^***^ (0.034)	0.229^***^ (0.020)	0.384^***^ (0.035)
Age	−0.020^***^ (0.001)	−0.036^***^ (0.001)	−0.001^**^ (0.001)	−0.002^*^ (0.001)
Marriage	−0.001 (0.003)	−0.001 (0.006)	−0.041^***^ (0.004)	−0.070^***^ (0.006)
Urban	0.009 (0.009)	0.021 (0.015)	0.068^***^ (0.010)	0.118^***^ (0.018)
Smoking	−0.015^***^ (0.003)	−0.025^***^ (0.005)	0.000 (0.003)	−0.001^**^ (0.005)
Edu	0.003^*^ (0.002)	0.006^*^ (0.003)	0.001 (0.002)	0.002 (0.003)
Work	0.002^***^ (0.000)	0.003^***^ (0.000)	−0.000 (0.000)	−0.001^*^ (0.000)
Income	0.013^***^ (0.003)	0.021^***^ (0.005)	0.014^***^ (0.003)	0.021^***^ (0.006)
Exercise	0.016^***^ (0.002)	0.029^***^ (0.004)	0.025^***^ (0.002)	0.043^***^ (0.004)
Fsize	0.008^**^ (0.004)	0.010 (0.007)	0.016^***^ (0.004)	0.026^***^ (0.006)
House	0.055^**^ (0.022)	0.092^**^ (0.036)	0.030 (0.021)	0.055 (0.036)
Pop	0.057 (0.109)	0.073 (0.184)	1.118^***^ (0.108)	1.934^***^ (0.185)
Hospital	0.022^*^ (0.014)	0.040^*^ (0.024)	0.019^**^ (0.014)	0.037^**^ (0.024)
*N*	20041	20041	20041	20041

Figures in parentheses are standard errors, ^*^, ^**^, ^***^ denote statistical significance levels at 10%, 5%, and 1% respectively.

The above shows that the construction of NFCs will significantly improve the residents' health, probably because the construction of NFCs is more to optimize the spatial layout of the city (48), the impact on the cost of housing is smaller, so the negative effect is offset, and ultimately, the net effect of the NFCs on the residents' health is positive.

However, the empirical model may have endogeneity, which make the estimation results have some bias. Therefore, the possible endogeneity problem will be dealt with in the following section using the instrumental variable method.

### 3.2 Instrumental variable regression

Table 5 shows the 2SLS estimation results. The first stage regression results show that there is a significant positive correlation between the instrumental variable and the dependent variable, which indicates that the more the number of NFCs construction in the province, the easier it is to carry out the construction of NFCs, which satisfies the correlation requirement of the instrumental variable. Further tests on instrumental variable showed that the *p*-value of the DWH endogeneity test was < 1%, indicating the presence of endogeneity. The Wald-F statistic was much >10, which can exclude the possibility of weak IVs. The second-stage regression coefficients show that the estimated coefficient for NFCs is significantly positive at least at the 5% level. The above results indicate that after dealing with the endogeneity problem, NFCs are still able to positively impact the residents' health.

**Table 5:** 2SLS regression.

	**(1) The first-stage regression**	**(2) The second-stage regression**	
	**Forestcity**	**Health**	**Mental**
Forestcity		0.2806^**^ (0.0683)	2.3475^***^ (0.2465)
Instrumental variable (NCities)	0.0283^***^ (0.0006)		
Control variables	Control	Control	Control
DWH endogeneity test *P*-value		7.0984 0.0077	66.8862 0.0000
Wald F statistic		2,494.60	2,494.60
N	17,219	17,219	17,219

Figures in parentheses are standard errors, ^*^, ^**^, ^***^ denote statistical significance levels at 10%, 5%, and 1% respectively.

### 3.3 Robustness tests

#### 3.3.1 Poisson model

The descriptive statistical analysis shows that the residents' health is non-negative integer. The differences between the mean (2.906) and the variance (1.407) of the physical health and the mean (26.359) and the variance (16.524) of the mental health are not too large, and the variances are smaller than means. To a certain extent, the assumption of “equal dispersion” of Poisson distribution is satisfied. Therefore, this section treats the residents' health as an unranked non-negative discrete variable, treats each health level equally. Poisson model were used to further ensure the robustness of the study results. The estimation results in Table 6 show that the estimated coefficients for NFCs are still significantly positive at the 1% level.

**Table 6:** Poisson regression.

	**(1) Poisson**	**(2)** **Poisson**	**(3) Poisson**	**(4)** **Poisson**
	**Health**	**Health**	**Mental**	**Mental**
Forestcity	0.051^***^ (0.008)	0.046^***^ (0.008)	0.021^***^ (0.003)	0.019^***^ (0.003)
Control variables	Control	Control	Control	Control
*N*	20,041	20,041	20,041	20,041

Figures in parentheses are standard errors, ^*^, ^**^, ^***^ denote statistical significance levels at 10%, 5%, and 1% respectively.

#### 3.3.2 Constructing new indicators

In this section, robustness tests were conducted after combining self-reported health into 3 and 2 categories. For mental health, a dummy variable of being depressed or not was used for robustness testing, the dummy variable for whether or not one is depressed was generated using the 95% quantile as a new criterion for depression measurement, based on a modification by Radloff (49), the developer of the Center for Streaming Depression Scale (CESD). The manners of definition are shown in Table 7. Based on the regression results in Table 8, it can be seen that the regression coefficients for the NFCs are all significantly positive at the 1%, once again validating the robustness of the baseline regression.

**Table 7:** Constructing new indicator definitions.

**Answer to question qp201**	**Health**	**Health 1**	**Health 2**	**CESD scores**	**Mental 1**
Pretty healthy	5	3	1	>19	1
Very healthy	4								
Relatively healthy	3	2			
Average	2	1	0	≤ 19	0
Unhealthy	1				

**Table 8:** Robustness test after constructing new indicators.

	**(1) Oprobit**	**(2)** **Probit**	** (3)** **Probit**
	**Health 1**	**Health 2**	**Mental 1**
Forestcity	0.104^***^	0.166^***^	0.147^***^
	(0.025)	(0.029)	(0.042)
Control variables	Control	Control	Control
*N*	20,041	20,041	20,041

Figures in parentheses are standard errors, ^*^, ^**^, ^***^ denote statistical significance levels at 10%, 5%, and 1% respectively.

#### 3.3.3 Replacing the dependent variable

This section uses other variables that measure health as the dependent variables for robustness testing. Specifically, the Body Mass Index (BMI) was used to replace self-reported health in the baseline regression, which was calculated as weight (kg)/height squared (m^2^). Memory was used to replace the CESD score in the baseline regression because memory is also a reflection of mental health, which corresponding to question qq501 in the CFPS 2018 questionnaire, “Can you remember the main things that happened to you during the week?.” Respondents were asked to choose between numbers 1 and 5 according to their actual situation, where “1” means that they can only remember a little, “2” means that they can only remember a few, “3” means that they can remember half, “4” means that they can remember most, and “5” means that they can remember all. Large numbers indicate a good state of mental health. The estimation results in Table 9 show that the coefficient of NFCs is significantly positive at the 1% level, which further indicates that the construction of NFCs does positively impact the residents' health.

**Table 9:** Robustness test after replacing dependent variable.

	**(1) OLS**	**(2)** **OLS**	**(3) Oprobit**	**(4)** **Oprobit**
	**BMI**	**BMI**	**memory**	**memory**
Forestcity	0.480^***^ (0.080)	0.475^***^ (0.077)	0.067^***^ (0.021)	0.060^***^ (0.022)
Control variables	No control	Control	No control	Control
*N*	20,041	20,041	20,041	20,041

Figures in parentheses are standard errors, ^*^, ^**^, ^***^ denote statistical significance levels at 10%, 5%, and 1% respectively.

#### 3.3.4 Excluding provinces without NFCs

Finally, the differences in unobservable factors such as human geography and living habits in the same province are relatively small considering. If the regression results are still robust after excluding provinces without NFCs, it proves that NFCs do promote the residents' health.

Therefore, Oprobit and Ologit regressions are conducted after excluding provinces without a NFC, leaving only provinces that contain at least one NFC. The estimation results in Table 10 are similar to the baseline regression, except that the coefficients are slightly smaller and the significance level is slightly lower, but still significantly positive at the 5% level. This further strengthens the robustness of the findings.

**Table 10:** Robustness test after excluding provinces without NFCs.

	**(1) Oprobit**	**(2)** **Ologit**	**(3) Oprobit**	**(4) Ologit**
	**Health**	**Health**	**Mental**	**Mental**
Forestcity	0.075^**^	0.112^**^	0.063^**^	0.110^**^
	(0.031)	(0.054)	(0.028)	(0.049)
Control variables	Control	Control	Control	Control
*N*	7,360	7,360	7,360	7,360

Figures in parentheses are standard errors, ^*^, ^**^, ^***^ denote statistical significance levels at 10%, 5%, and 1% respectively.

In summary, it can be seen that the results of a series of robustness tests indicate that the results of the baseline regression in this article are relatively robust.

### 3.4 Influential mechanisms

On the basis of the previous article, the influential mechanisms of NFCs on residents' health will be empirically tested next. First, on the basis of the baseline regression, this article adds the interaction term of the *forestcity* and the health risk exposure level variables, and the interaction term of the *forestcity* and the residents' exercise behaviors variables, respectively. Referring to the practices of the existing studies, the concentration of air pollutants SO_2_ (*SO*_2_) and soot (*soot*) are used to measure the health risk exposure level. The frequency (*qp701*) and length (*qp702*) of exercise of the residents in a week are used to characterize the residents' exercise behaviors.

#### 3.4.1 Reducing health risk exposure

In the baseline model, the *forestcity* × *SO*_2_, *forestcity* × *soot* were added. The regression results are shown in Table 11, and the coefficients of the interaction terms are all significantly negative. This suggests that NFCs reduce residents' health risk exposure by reducing the concentration of air pollutants SO_2_ and soot, thus improving residents' physical and mental health. On the one hand, reducing health risk exposure will reduce the incidence of various diseases (14); on the other hand, good air quality will alleviate residents' anxiety and despair, thus promoting residents' psychological health (19). Therefore, the above analyses show that the construction of NFCs can improve residents' health by reducing health risk exposure.

**Table 11:** Mechanism analysis 1.

	**(1)**	**(2)**	**(3)**	**(4)**	**(5)**	**(6)**
	**Health**	**Health**	**Health**	**Mental**	**Mental**	**Mental**
Forestcity	0.140^***^ (0.023)	0.196^***^ (0.044)	0.157^***^ (0.042)	0.137^***^ (0.020)	0.149^***^ (0.038)	0.165^**^ (0.037)
Forestcity × so2		−0.008^**^ (0.003)			−0.005^*^ (0.003)	
Forestcity × soot			−0.002^**^ (0.003)			−0.008^***^ (0.003)
Control variables	Control	Control	Control	Control	Control	Control
N	20,041	14,940	14,940	20,041	14,940	14,940

Figures in parentheses are standard errors, ^*^, ^**^, ^***^ denote statistical significance levels at 10%, 5%, and 1% respectively.

#### 3.4.2 Promoting residents' health behavior

In the baseline model, the interaction term of *forestcity* and residents' exercise behavior were added. The regression results are shown in Table 12, the coefficients of the interaction terms are significantly positive, indicating that the construction of NFCs can improve the health level of residents by positively promoting residents' exercise behaviors. The environment in most modern cities has a great impact on the nervous system of our brain, which may make us feel tired, nervous and depressed. In contrast, plants and flowers in nature can emit some volatile organic compounds that can attract people to increase jogging and engage in outdoor exercise, and these activities can effectively enhance the body's immunity and the function of various systems, making us healthier.

**Table 12:** Mechanism analysis 2.

	**(1)**	**(2)**	**(3)**	**(4)**	**(5)**	**(6)**
	**Health**	**Health**	**Health**	**Mental**	**Mental**	**Mental**
Forestcity	0.140^***^ (0.023)	0.111^***^ (0.031)	0.135^***^ (0.024)	0.137^***^ (0.020)	0.120^***^ (0.027)	0.135^***^ (0.021)
Forestcity × qp701		0.009^**^ (0.007)			0.010^**^ (0.006)	
Forestcity × qp702			0.002^*^ (0.002)			0.003^**^ (0.001)
Control variables	Control	Control	Control	Control	Control	Control
N	20,041	11,883	11,883	20,041	11,883	11,883

Figures in parentheses are standard errors, ^*^, ^**^, ^***^ denote statistical significance levels at 10%, 5%, and 1% respectively.

### 3.5 Heterogeneity analysis

The mechanism analysis found that NFCs improve residents' health mainly by reducing health risk exposure and promoting residents' health behaviors, and this effect may be differentiated for populations in different regions. This article uses group regression to test for heterogeneity, separately testing the differences in the health promotion effects of NFCs on residents between urban and rural areas, older adult and younger adult, and residents with different income levels.

#### 3.5.1 Heterogeneity between urban and rural areas

The problem of disparities between regions is of great concern, given the different natural resource endowments, the degree of economic development and the industrial structure of different regions of China. As an important feature of China's social structure, urban-rural co-development is of great significance in building a harmonious society, promoting social stability and enhancing the health of residents. In this study, the National Bureau of Statistics (NBS) data was used as the basis for dividing urban and rural areas and analyzing the differences in the impact of NFCs on residents' health from the perspective of urban-rural differences. The results in Table 13 show that the construction of NFCs can significantly improve the health level of urban and rural residents, but the impact on the health level of urban residents is significantly higher than that of rural residents. On the one hand, the widening gap in income, industry and other economic aspects is not conducive to the balanced development of urban and rural residents in terms of health, which can inhibit the subjective sense of wellbeing of rural residents. On the other hand, there are real problems such as unbalanced development, insufficient advancement, and mismatch between supply and demand in forest cities, and the non-equalization of urban and rural public services affects the physical activity status of residents in rural areas, and the probability of urban residents engaging in physical activity is greater.

**Table 13:** Heterogeneity analysis.

	**(1) Rural**	**(2) Urban**	**(3) The young**	**(4) Older adult**	**(5) Lower-income group**	**(6) Higher-income group**
	**Health**	**Health**	**Health**	**Health**	**Health**	**Health**
Forestcity	0.081^**^ (0.036)	0.184^***^ (0.034)	0.131^***^ (0.028)	0.151^***^ (0.049)	0.173^***^ (0.027)	0.085^*^ (0.058)
Control variables	Control	Control	Control	Control	Control	Control
*N*	10,779	9,187	14,905	5,136	16,846	3,195

Figures in parentheses are standard errors, ^*^, ^**^, ^***^ denote statistical significance levels at 10%, 5%, and 1% respectively.

#### 3.5.2 Heterogeneity of older and younger populations

Age groupings ≥60 years are considered as the older adult, otherwise they are considered as the younger adult. The results in Table 13 show that the coefficient for older adult is greater than that of the younger adult and they are both statistically significant. This may be due to the fact that older people enjoy their retirement and have more leisure time to enjoy the infrastructural benefits of NFCs, such as green parks.

#### 3.5.3 Heterogeneity of income levels

The income grouping is individual income ≥28,228 yuan (per capita income of Chinese residents in 2018) is the high-income group, otherwise it is the low-income group. The results in Table 13 show that the regression coefficient value of the low-income group is larger than that of the high-income group. More notably, the effect of NFCs on the low-income group is more significant, which suggests that NFCs bring more of a public welfare, probably because the construction of parks, green spaces and other infrastructures in NFCs is equitably accessible to Chinese residents, so there is no environmental justice problem, indicating that the construction of forest cities to a certain extent offsets the health gap brought about by the income gap.

## 4 Discussion

This article empirically studies the relationship between NFCs and residents' health, and mainly obtains the following conclusions: first, the construction of NFCs can significantly improve the health level of residents, which is manifested in both physiological and psychological aspects, and the results of the instrumental variable regression still support the above conclusions; second, the construction of NFCs mainly improves the health level of residents by reducing the exposure to health risks and promoting the healthy behaviors of the residents; third, the impact of NFCs on the health of the residents exists heterogeneity between different groups, and the NFCs have greater effect on the improvement of the health level of the people who reside in the city, are older, and have a lower income.

The innovation of this article is that there is no literature that directly focuses on the impact of NFCs on residents' health. In this article, based on the CFPS2018 data and matched with the 2016–2018 list of NFCs, we finally obtained 20,041 samples to study the impact of NFCs on residents' health and its mechanisms. In addition, we analyzed the heterogeneity of the impact of NFCs on the health of different groups. The possible marginal contributions are mainly reflected in the following four aspects: firstly, it is verified that the construction of NFCs improves the residents' health from both physical health and mental health levels at the same time. Secondly, the mechanism of the impact of the NFCs on residents' health was clarified, especially assessing the effectiveness of the construction of NFCs in terms of infrastructure such as parks and green spaces, i.e., promoting residents' exercise behavior. Third, it was further tested that the construction of NFCs brings a public welfare that promotes the public health of the general public, and its impact on the health of the residents is a slow, general and long-term impact effect. Fourth, in terms of research methodology, one of the robustness tests tentatively considers residents' health as a non-negative discrete variable without ordering, and treats each level of residents' health equally, and enriches the research by using Poisson regression models to further test the robustness of the article's results.

More notably, the heterogeneity analyses in this article find that NFCs have a more significant impact on residents with lower incomes, suggesting that NFCs are a public good, which to some extent counteracts the “health-income stratification phenomenon.” With regard to foreign cases, access to green space is an environmental justice issue, and scholars have described how inequitable access to urban tree cover may lead to health disparities among different populations (50). China's basic national conditions, however, dictate that the likelihood of such a situation exists is low, which is aptly confirmed by the results of this article.

In addition, our study has some limitations. Firstly, due to the limitation of cross-sectional data, we did not discuss the long-term impact of NFCs on residents' health. Second, due to space limitations, this study did not explore the health impacts of specific ecological products of NFCs. In the future, research on quantitative value assessment methods for the effectiveness of NFCs can be carried out around the gross ecosystem product and the realization of the value of ecological products in forest cities.

## 5 Conclusions and suggestions

The construction of NFCs not only plays an important role in improving the human environment, improving the economy, and enhancing the city's image, but also has an increasing impact on individual behavior. In this article, we match the list of NFCs from 2016–2018 with the data from the China Family Panel Studies data in 2018 (CFPS2018), and empirically verify that NFCs significantly promote residents' health. This proves that NFC construction has public health value. Therefore, to further support the realization of the Healthy China strategy, we suggest that the government should pay attention to the construction of NFCs and accelerate the promotion of the formation of a new situation of health for all.

According to the results of the mechanism analysis, reducing health risk exposure and promoting residents' exercise behavior are the two mechanisms by which NFCs promote residents' health. It shows that the construction of NFCs has not only achieved results in afforestation, bringing ecological benefits to residents; but also achieved new results in recreational places such as parks and green spaces, bringing infrastructural benefits to residents. Therefore, in the future, in the construction planning of NFCs, in addition to continuing to increase afforestation, the construction of parks, green spaces and other recreational places should also be placed in an important position. Maximize public health benefits while avoiding the urban green space paradox.

According to the results of the heterogeneity analyses, compared to urban residents, NFCs have a lesser role in promoting the health of rural residents. Considering the low level of primary healthcare services and the inadequate service facilities in rural areas, we suggest that more support should be given to rural areas so as to improve their overall wellbeing. In addition, we found that NFCs have a smaller impact on younger adult than on the older adult, suggesting that young people have a slightly lower use of infrastructure such as parks and green spaces. Therefore, society and industry should strengthen guidance for young people to use more recreational green spaces in general to enhance their physical fitness and reduce their reliance on mobile phones and computers.

## Data availability statement

The original contributions presented in the study are included in the article/supplementary material, further inquiries can be directed to the corresponding author.

## Author contributions

HX: Funding acquisition, Methodology, Project administration, Resources, Supervision, Writing—review & editing. CY: Conceptualization, Data curation, Formal analysis, Investigation, Software, Writing—original draft. XT: Supervision, Validation, Visualization, Data curation, Resources, Writing—review & editing.
